# Porcine Parvovirus 2 Is Predominantly Associated With Macrophages in Porcine Respiratory Disease Complex

**DOI:** 10.3389/fvets.2021.726884

**Published:** 2021-08-13

**Authors:** April Nelsen, Chun-Ming Lin, Ben M. Hause

**Affiliations:** ^1^Department of Veterinary and Biomedical Sciences, South Dakota State University, Brookings, SD, United States; ^2^Animal Disease Research and Diagnostic Laboratory, South Dakota State University, Brookings, SD, United States

**Keywords:** porcine parvovirus, porcine respiratory disease complex, tissue microarray, metagenomic sequencing, *in situ* hybridization

## Abstract

Porcine respiratory disease complex (PRDC) is a significant source of morbidity and mortality, manifested by pneumonia of multiple etiologies, where a variety of pathogens and environment and management practices play a role in the disease. Porcine reproductive and respiratory syndrome virus (PRRSV), influenza A virus (IAV), and porcine circovirus 2 (PCV2) are well-established pathogens in PRDC. Porcine parvovirus 2 (PPV2) has been identified in both healthy and clinically diseased pigs at a high prevalence worldwide. Despite widespread circulation, the significance of PPV2 infection in PRDC and its association with other co-infections are unclear. Here, PPV2 was detected in the lung tissue in 39 of 100 (39%) PRDC-affected pigs by quantitative polymerase chain reaction (qPCR). Using *in situ* hybridization (ISH) in conjunction with tissue microarrays (TMA), PPV2 infection was localized in alveolar macrophages and other cells in the lungs with interstitial pneumonia in 28 of 99 (28.2%) samples. Viral load tended to correlate with the number of macrophages in the lungs. Assessment of the frequency, viral titers, and tissue distributions showed no association between infection of PPV2 and other major viral respiratory pathogens. In one-third of the PPV2-positive samples by qPCR, no other known viruses were identified by metagenomic sequencing. The genome sequences of PPV2 were 99.7% identical to the reference genomes. Although intensive intranuclear and intracytoplasmic signals of PPV2 were mainly detected in alveolar macrophages by ISH, no obvious virus replication was noted in *in vitro* cell culture. Together, these results suggest that PPV2 is associated, but may not be the sole causative agent, with PRDC, warranting the control and prevention of this underdiagnosed virus.

## Introduction

Porcine parvovirus 2 (PPV2), formally *Ungulate tetraparvovirus 3*, belongs to the *Tetraparvovirus* genus, Parvoviridae family (1). PPV2 is a small, non-enveloped, icosahedral virus consisting of an ~5–6 kb linear, single-stranded DNA (ssDNA) genome (1, 2). The prototype of porcine parvovirus, porcine parvovirus 1 (PPV1), is a well-known pathogen that causes reproductive failure (3). Porcine parvoviruses are frequently identified as co-infections with other bacterial and viral pathogens in diseases such as porcine respiratory disease complex (PRDC) and porcine circovirus-associated disease (PCVAD) (4, 5). The clinical significance of PPV1 has been well-described, while for emerging parvoviruses such as PPV2, the pathological consequences of infection are poorly understood (3). PPV2 was first identified in Myanmar in 2001; however, a retrospective study of samples collected from several European countries detected PPV2 as early as 1998 in archived porcine tissues (6, 7).

Epidemiological studies of PPV2 have reported varying prevalence rates worldwide. In clinically healthy pigs, a high prevalence of PPV2 was detected in oral fluids (48.7%) and serum (53.9%) in Poland between 2015 and 2017 (8). Likewise, PPV2 was detected in 78.0 and 70.5% of tonsils and hearts, respectively, in Germany (9). The tonsils of healthy slaughterhouse pigs collected in Japan (2010) and Thailand (2011) identified 69 out of 120 (58%) and 66 out of 80 (83.0%) positive for PPV2, respectively (10, 11). In sick pigs, PPV2 was detected in 8.8% swine serum samples in a 2006 outbreak of “high fever” observed in swine with porcine reproductive and respiratory syndrome (PRRSV) and/or post-weaning multisystemic wasting syndrome (PWMS) in China (6). In Japan, PPV2 was identified in 69 out of 69 (100%) sick domestic pig tonsils submitted for diagnostic testing in 2010 (10). A study in the United States detected PPV2 in 100 out of 483 (20.7%) randomly selected lung tissues from routine diagnostic cases of various diseases (12). A retrospective study of Hungarian swine herds from 2006 to 2011, all positive for porcine circovirus 2 (PCV2), identified PPV2 in 4 out of 38 (10.5%) lung samples (2).

A more recent retrospective study using histopathology scoring showed that PPV2 was significantly associated with respiratory pathogenesis and disease, in particular interstitial pneumonia (IP) and bronchointerstitial pneumonia (BIP) (7). Concurrent infections of PPV2 and PCV2 were identified in 90 out of 695 (12.9%) tissue samples, with PPV2 identified in association with PCV2 in PRDC and PCVAD by latent class analysis (7). PPV2 has been identified in cells morphologically resembling macrophages and lymphocytes in the lung tissues of 2–3-month-old dead pigs by *in situ* PCR and *in situ* hybridization (ISH) in lesions of systemic perivascular inflammation in pigs with poor growth (1, 13). To date, studies of the infection pathology of PPV2 are limited.

Porcine respiratory disease complex is the result of multiple viral and/or bacterial co-infections. PCV2, PRRSV, and IAV are recognized as the main causative agents (14). While PPV2 is an emerging virus reported as highly prevalent in a variety of porcine tissues from both healthy and sick pigs, it has not yet been included in regular diagnostic panels for PRDC. As part of a routine diagnostic investigation, metagenomic sequencing was performed on a swine lung tissue with interstitial pneumonia that was PCR negative for IAV, PRRSV, PCV2, and *Mycoplasma hyopneumoniae* in our diagnostic laboratory. PPV2 was identified in the absence of other swine viruses and clinically significant bacteria, leading us to here investigate the role of PPV2 in PRDC.

## Materials and Methods

### Sample Selection

All porcine respiratory cases submitted to the Animal Disease Research and Diagnostic Laboratory at South Dakota State University (ADRDL-SDSU) from 2019 to 2020 were screened for archived formalin-fixed paraffin-embedded (FFPE) lung tissues. The samples included lung tissues (*n* = 124) and variably contained tissues from the liver, kidney, lymph node, or the spleen, embedded in the same FFPE block.

Retained frozen tissue samples submitted to ADRDL-SDSU between May 2020 and September 2020–January 2021 were selected from porcine respiratory diagnostic submissions that included lung tissue. The tissue (*n* = 100) was homogenized and stored at −80°C.

Case data, including the age of the pigs and clinical signs, were recorded. Client-requested diagnostic testing results, including PCR for IAV, PRRSV, PCV2, porcine circovirus 3, and *M. hyopneumoniae* and bacterial isolation, were collected as available.

Pigs were separated into six groups to determine the PPV2 prevalence at different ages: fetuses (<1 day), suckling pigs (1 day−3 weeks), nursery pigs (3–8 weeks), grow-finish pigs (8–25 weeks), mature pigs (>25 weeks), and unknown.

### Quantitative PCR

Nucleic acids were extracted from homogenized tissue with the QIAamp^®^ Viral RNA Mini Spin (QIAGEN, Hilden, Germany) as per the manufacturer's instructions. For the FFPE samples, QIAamp^®^ DNA FFPE Advanced Kit and QIAamp^®^ DNA FFPE Advanced UNG Kit (QIAGEN, Hilden, Germany) were used. A previously designed PPV2 TaqMan^®^ assay was used (12). A master mix consisting of TaqMan^®^, PPV2 primer/probe, and RNase-free water was added to the sample and ran on the 7500 FAST Real-Time PCR System (Applied Biosystems, Waltham, MA, USA) with an initial 20-s hot start at 95°C followed by 40 cycles of 15 s at 95°C and 1 min at 60°C.

### Construction of the Tissue Microarray and Histopathology Examinations

To assess the pathology of PPV2 infection in the lungs, 100 FFPE blocks, including 27 samples where the lung homogenates were positive for PPV2 by quantitative PCR (qPCR), 23 samples where the lung homogenates were negative for PPV2 by qPCR, and 49 randomly selected cases with unknown PPV2 status, were selected from the FFPE tissue archive. One tissue was mistakenly classified as lung and removed from the study, for a final total of 99 tissue cores used in the study. Tissue cores with a diameter of 5 mm, containing at least seven intact bronchioles and adjacent pulmonary parenchyma, were extracted from donor lung blocks and transferred to a recipient paraffin block with a random distribution (AMSBIO LLC, Cambridge, MA, USA). Five tissue microarray (TMA) recipient blocks, each containing 20 cores, were constructed. Serial sections of TMA blocks were made for routine hematoxylin–eosin (HE), immunohistochemistry (IHC), and ISH staining, as described below. Finally, the TMA slides were read by a pathologist with the case number blinded. They were also digitized using a DP74 camera (Olympus, Tokyo, Japan) at ×200 magnification. Signals of ISH and IHC were semi-quantified using the Olympus cellSens Cell Count & Measurement module.

### Porcine Parvovirus 2 *in situ* Hybridization

*In situ* hybridization was performed using a commercialized RNAscope^®^ system [Advanced Cell Diagnostics (ACD), Newark, CA, USA] according to the manufacturer's instructions. The proprietary PPV2 probe (cat. #899191) from the ACD targeting viral capsid gene was used. A positive (RNAscope^®^ Probe Ss-PPIB, cat. #428591) and a negative (RNAscope^®^ Negative control Probe-DapB, cat. #310043) probe were used as internal controls. Briefly, the TMA slides were incubated for 1 h at 60°C in a HybEZ™ Oven from ACD. The slides then underwent deparaffinization with two rounds of 5 min in xylene followed by two rounds for 1 min of 100% ethanol. Pretreatment of the FFPE slides included 10 min at room temperature with RNAscope^®^ hydrogen peroxide, 15 min in a Hamilton Beach 5.5 Quart 37530A Digital Food Steamer with RNAscope^®^ 1X Target Retrieval Reagent, and 30 min at 40°C in the HybEZ™ Oven with RNAscope^®^ Protease Plus. The following steps were completed according to the RNAscope^®^ Assay using the RNAscope^®^ 2.5 HD Detection Reagent—RED. The slides were incubated for 2 h in the HybEZ™ Oven at 40°C for 2 h with the PPV2 ORF2 messenger RNA (mRNA) probe provided by ACD. The slides underwent four rotating amplification steps of 30 min at 40°C followed by 15 min at 40°C. Two additional amplification steps followed at room temperature, one for 30 min and one for 15 min. Signal was detected with Fast RED with a 10-min room temperature incubation. The slides were counterstained with 50% Gill's hematoxylin I and mounted with EcoMount.

### Nuclease Treatment

To differentiate between ISH detection of PPV2 mRNA as opposed to the genome of PPV2 in the lungs, serial sections from one case with intense PPV2 ISH staining were treated with either Ambion^™^ DNase I (RNase-free) or RNase ONE^™^ Ribonuclease for 30 min at 37°C before the hybridization procedure. The working concentration of DNase I was 0.04 U/μl and that of RNase ONE^™^ was 0.2 U/μl.

### Immunohistochemistry

Immunohistochemistry was performed on TMA slides to detect the swine pathogens PRRSV (SDOW17/SR30, and SDSU), IAV (HB65 and SDSU), and PCV2 (ISU-4; Iowa State University). Phenotyping of inflammatory cells was performed with the cell markers CD3 (Dako, Carpinteria, CA, USA), CD79a (Dako), and Iba-1 (ThermoFisher, Waltham, MA, USA) for T lymphocytes, B lymphocytes, and macrophages, respectively, as described in a previous study (15). Antigen retrieval was conducted by heating in a microwave (1,100 W) for 2 min and 10 s in pH 6.0 citrate buffer. Subsequently, the slides were loaded onto Dako Autostainer-Universal Staining System. The protocol was carried out with rabbit-specific HRP/DAB Detection IHC Detection Kit—Micro-polymer (Abcam, Cambridge, UK) according to the manufacturer's instructions.

### Metagenomic Sequencing

Metagenomic sequencing was performed on frozen tissue samples that were qPCR positive for PPV2 but negative (or data not provided) for IAV, PRRSV, PCV2, and *M. hyopneumoniae* to identify co-infections present with PPV2 in respiratory disease submissions. Metagenomic sequencing was performed as previously described (16, 17). Briefly, clarified tissue homogenate was digested with a cocktail of nucleases followed by extraction using the QIAamp^®^ Viral RNA kit. First-strand synthesis using the SuperScript^™^ III First-Strand Synthesis Kit was performed on the nucleic acid extracts using barcoded oligonucleotides containing 3′ random hexamers followed by digestion with RNase H. Second-strand synthesis using Sequenase was performed next. Complementary DNA (cDNA) was purified with the GeneJET Gel Extraction and DNA Cleanup Micro Kit (ThermoFisher) and subsequently amplified by PCR using primers specific to the integrated barcode. Amplified DNA was purified using the GeneJET Gel Extraction and DNA Cleanup Micro Kit. The concentration of purified DNA was measured on a Qubit spectrophotometer and diluted to 0.2 ng/μl. Sequencing libraries were prepared with a Nextera XT kit (Illumina, San Diego, CA, USA). DNA sequencing was performed with an Illumina MiSeq using paired 151-bp chemistry. Sequencing reads were trimmed with onboard software and imported into CLC Genomics Workbench 20 (QIAGEN), where they were assembled into contigs using the *de novo* assembler. Contigs were analyzed by BLASTX using the BLAST2GO Plug-in to identify reads similar to known swine viruses.

### Genetic and Phylogenetic Analysis

To investigate the relationship of the PPV2 sequenced here with the previously determined PPV2 genomes, 28 PPV2 genomes determined from a broad geography from 2012 to 2018, along with representative Parvovirinae, were downloaded from GenBank. Whole-genome phylogenetic analysis was performed using the best-fitting K2+G (Kimura two-parameter) model of evolution with 1,000 bootstrap replicates as implemented in the MEGA X software program. Complete PPV2 coding DNA sequences determined directly from two clinical samples were submitted to GenBank under the accession numbers MW883398 and MW883399.

### Virus Isolation

Nine clinical samples that were positive for PPV2 by qPCR (Ct range of 16.9–32.8) were filtered through a 0.22-μm membrane filter, and 100 μl filtrate was used to inoculate porcine alveolar macrophages (PAMs) collected from pigs housed at ADRDL-SDSU for an unrelated study. Four of the nine clinical samples were also positive for PRRSV and/or IAV.

PAMs were seeded into a 24-well plate at 1 × 10^6^ cells/well in RPMI with 10% fetal bovine serum (FBS) and 1% penicillin–streptomycin for 24 h. Virus isolation was attempted by adsorption of both the inoculum followed by washing and refeeding and with the inoculum left in the cell culture media.

For virus isolation on PAM cells, 100 μl sample filtrate was added to the cells, incubated at 37°C, and the supernatants passed on day 5 post-infection for up to four passes and harvested for analysis by qPCR for PPV2. Cells were harvested on day 5 post-infection for analysis by ISH for PPV2. Alternatively, virus isolation was performed with the PPV2 inoculum removed from PAMs 24 h following seeding. Of the sample filtrate, 100 μl was adsorbed onto cells for 1 h in a 24-well plate at 37°C. The supernatant was removed and the media replaced to incubate at 37°C for 5 days. Cells were harvested on day 5 post-infection for analysis by ISH for PPV2.

### Statistical Analysis

Statistical analyses were carried out with the Statistical Analysis System (SAS) program. A *P* < 0.05 was considered statistically significant. However, a *P* < 0.2 was considered a tendency, given the high degree of variability associated with the nature of diagnostic submissions (18). Pearson's chi-square test was used to determine the frequencies in independent events. Peterson's correlation was applied on the qPCR Ct values of PPV2, PRRSV, IAV, PCV2, and *M. hyopneumoniae*, the PPV2 ISH signal count, and the age of swine. Multiple linear regression analysis was performed using the load of each pathogen as variables to predict the number of inflammatory cell populations in PRDC.

## Results

### Prevalence of PPV2 and Other Respiratory Pathogens by qPCR

Thirty-nine out of 100 (39%) lung tissue homogenates were qPCR positive for PPV2. Of the 124 FFPE sections from porcine respiratory cases, nine were qPCR positive for PPV2, giving a 7% positivity rate. Due to the apparent decreased sensitivity of PPV2 detection in FFPE tissues by qPCR, prevalence and statistical analyses were only performed on tissue homogenates.

PPV2 was detected in each of the six age groups, with the highest prevalence (63.6%) of cases in the age group of grow-finish pigs (8–25 weeks) (Table 1). In addition, there was a negative correlation between the Ct value of PPV2 and the age of pigs (*r* = −0.28, *P* < 0.05).

**Table 1:** Prevalence of porcine parvovirus 2 (PPV2) detected by quantitative PCR (Ct range of 13.88–33.66; Ct median of 25.76) in different age groups.

**Age group**	**No. of samples** **(*n* = 100)**	**PPV2-positive cases** **(%)**
Fetuses (<1 day)	10	1 (10)
Suckling pigs (1 day−3 weeks)	10	2 (20)
Nursery pigs (3–8 weeks)	36	12 (33.3)
Grow-finish pigs (8–25 weeks)	11	7 (63.6)
Mature pigs (>25 weeks)	4	1 (25)
Unknown	29	16 (55.2)

Lung tissue homogenates showed prevalence rates of 32.9% (27/82), 28.2% (20/71), 22.2% (6/27), and 33.3% (13/39) for PRRSV, IAV, PCV2, and *M. hyopneumoniae*, respectively (Supplementary Table 1). Among the samples also tested for other pathogens, PPV2 was co-infected in 13.4% (11/82), 12.5% (9/72), 10.7% (3/28), and 22.5% (9/40) cases with PRRSV, IAV, PCV2, and *M. hyopneumoniae*, respectively. Although co-infections were commonly observed, chi-square analysis and Fisher's exact test found that infections of PPV2 and other pathogens were independent events. There was no correlation between the Ct values of PPV2 and other pathogens. However, a significant positive correlation between the titers of PCV2 and *M. hyopneumoniae* was noted (*r* = 0.65, *P* < 0.05).

### Detection of PPV2 and Other Respiratory Pathogens by ISH and IHC in Tissue Microarrays

**Figure 1:**
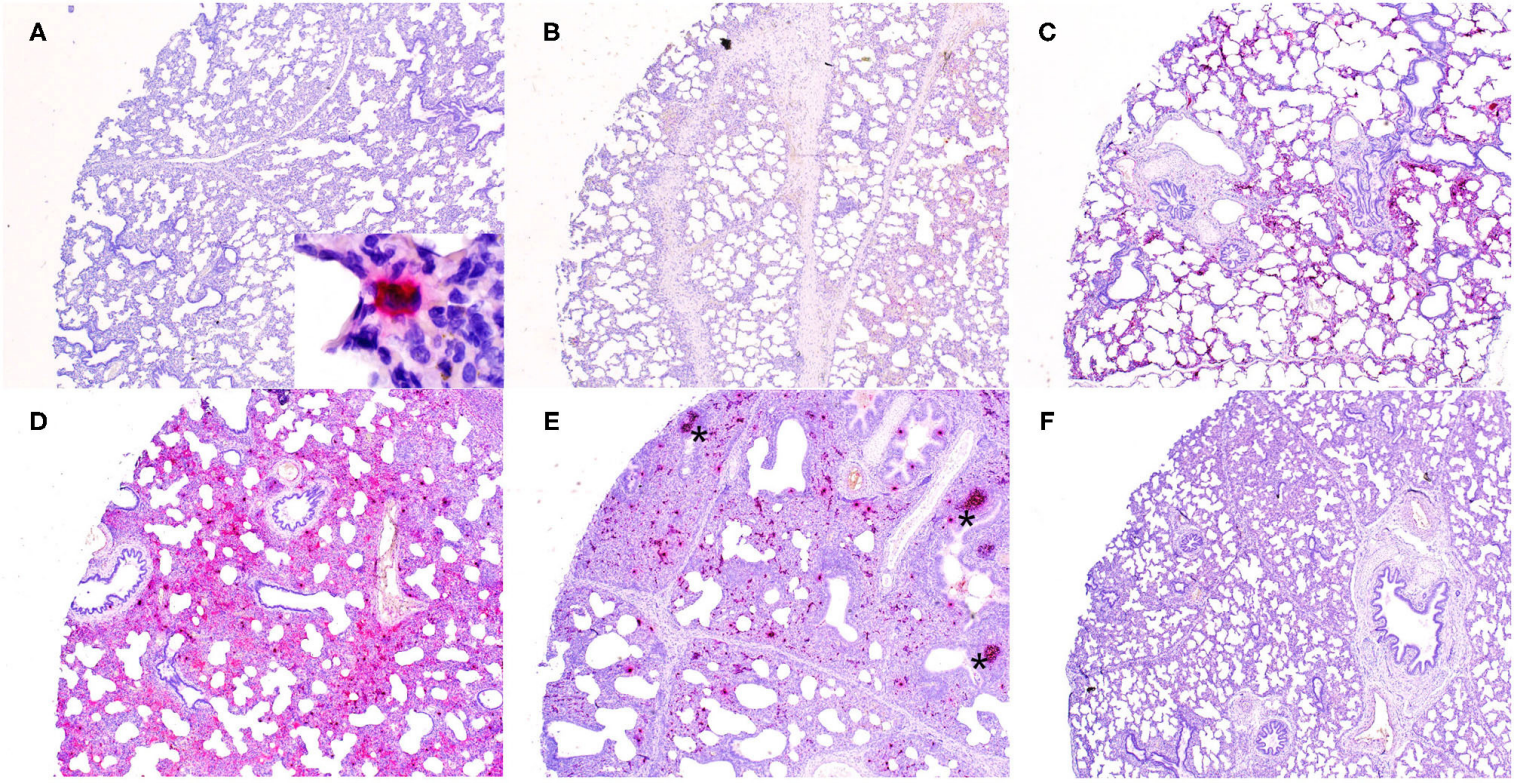
Detection of porcine parvovirus 2 (PPV2) nucleic acid in the lungs obtained from porcine respiratory disease complex-affected pigs by *in situ* hybridization (ISH, ×40). The ISH signals of PPV2 ranged from a focal single individual cell **(A)** to multifocal **(B,C)** to diffuse **(D,E)**. No signal was detected in a tissue core obtained from a PPV2 qPCR-negative case **(F)**. Note that the signals of PPV2 were mainly detected in the pulmonary interstitium and activated lymphoid follicles (*asterisk*) in peribronchial lymphoid tissues **(E)**.

**Figure 2:**
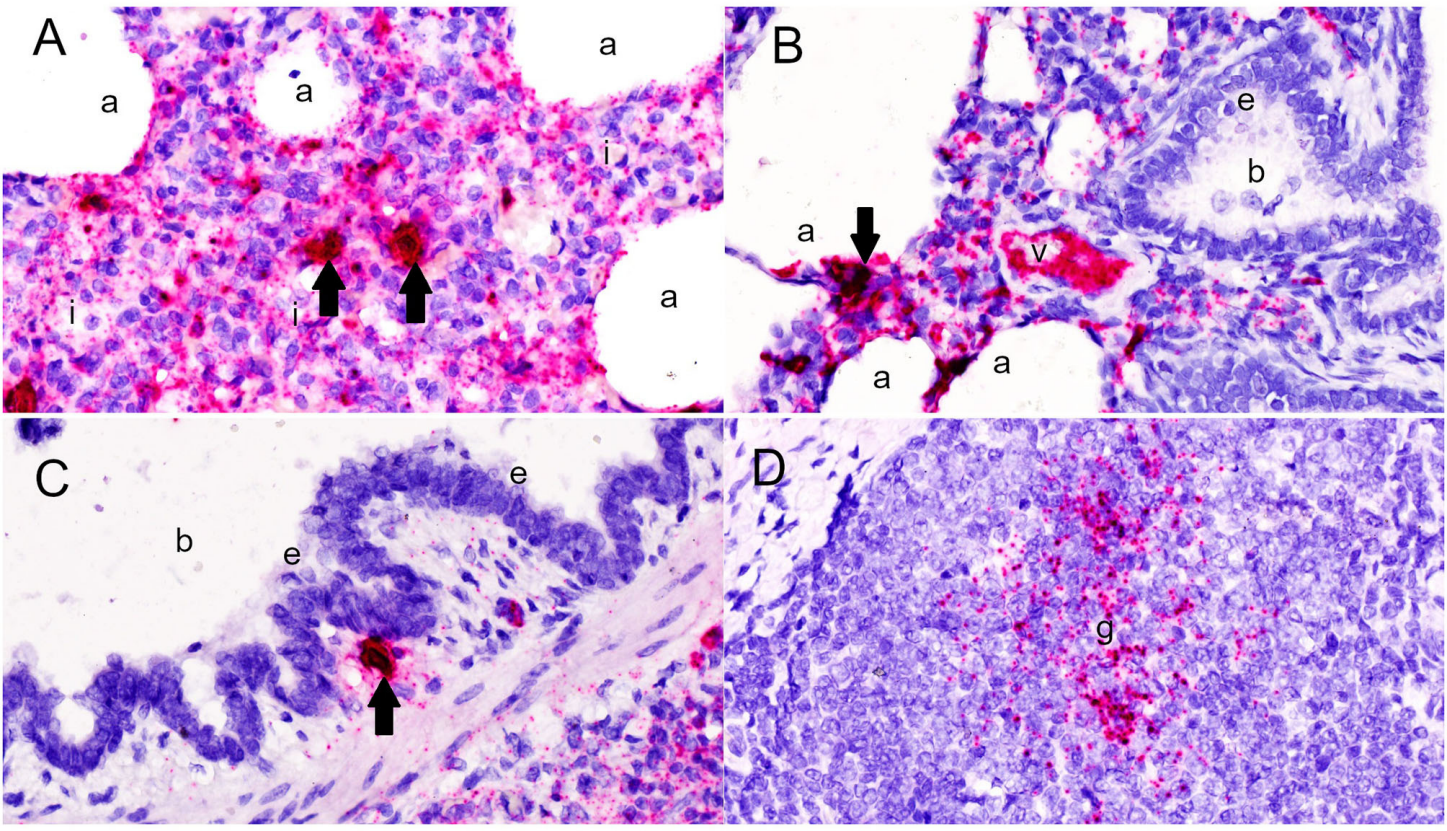
Detection of porcine parvovirus 2 (PPV2) nucleic acid in the lungs by *in situ* hybridization (ISH, ×600). **(A,B)** The ISH signals of PPV2 were detected as pinpoints in the cytoplasm of monocytic cells expanding the alveolar interstitium (*i*), as well as pneumonocytes and alveolar macrophages lining the alveoli (*a*) and endothelial cells lining the small blood vessels (*v*). **(C)** Intensive intracytoplasmic and intranuclear signals of PPV2 were detected in large cells resembling macrophages or dendritic cells (*arrows*). PPV2 signals were also detected in round cells around the bronchus (*b*), but not the bronchial epithelium (*e*). **(D)** Aggregation of the PPV2 ISH signals in the germinal center (*g*) of the lymphoid follicle in peribronchial lymphoid tissues.

Microscopically, varying intensities of PPV2 ISH signals with distribution patterns ranging from a single or a few individual cells (Figure 1A) to multifocal, extensive patches (Figure 1B) to diffuse (Figures 1C–E) were detected in 28 of 99 tissue cores, while no signal was detected in the other 71 cores on the same TMA slides (Figure 1F). PPV2 nucleic acid was hybridized as numerous pinpoints in the cytoplasm of round cells, morphologically compatible with macrophages and some lymphocytes (Figure 2A). PPV2 was also detected in pneumonocytes, vascular endothelial cells, and, occasionally, fibroblast-like cells. Frequently, intensive intracytoplasmic and intranuclear PPV2 ISH signals, diffusely obscuring the entire cellular details, were observed in large round cells, morphologically compatible with alveolar and infiltrating macrophages (Figure 1A, insert, and Figures 2A–C). The distribution of the PPV2 ISH signals was consistent with interstitial pneumonia, characterized by macrophages and T lymphocytes expanding the alveolar septa, activated alveolar macrophages and type II pneumonocytes lining the alveolar walls (Figure 2A), and hypertrophic vascular endothelial cells lining the vessel capillaries (Figure 2B).

Although pneumonia was commonly complicated by neutrophilic inflammation centering in airways (neutrophilic bronchopneumonia) in 36% of the tissues cores, no PPV2 nucleic acid was detected in the bronchial and bronchial epithelium (Figure 2C). However, intensive signals of PPV2 nucleic acid were clustered at cells morphologically resembling immunoblasts, dendritic cells, and macrophages in the germinal centers of peribronchial lymphoid tissues (PBLTs) (Figures 1E, 2D) and were also detected sporadically in some lymphocytes, macrophages, and dendritic cells (Figure 2C) infiltrating and scattering around the inflamed bronchi and bronchioles.

Overall, the number of PPV2 ISH signals counted by image software correlated significantly with the Ct values of the PPV2 genome detected by qPCR (*r* = −0.64, *P* < 0.001). All the cases with PPV2 qPCR Ct values <20 (*n* = 6) showed a diffuse and intensive positive signal of PPV2 in TMA cores (Figures 1D,E). With the exception of two cases that had few PPV2 ISH-positive signals detected in the germinal centers of PBLTs, no ISH signal was detected in cases with Ct value >30 (Figure 1F).

Antigens of PRRSV, IAV, and PCV2 were detected in 13, 1, and 0 tissue cores, respectively, by routine IHC in serial TMA sections. Among them, concurrent positive signals of PPV2 nucleic acid and PRRSV antigen were noted in three tissue cores; however, co-localization of PPV2 ISH and PRRSV IHC signals was not obviously observed in serial TMA sections.

The morphological diagnoses of pneumonia and the interpretation of the PPV2 ISH signal distributions were further supported by phenotyping of immune cells in the lungs. Semi-quantification of cell markers revealed a positive correlation between the number of CD3-positive T cells and IBA 1-positive macrophages (*r* = 0.36, *P* < 0.05), representing monocytic pneumonia. CD79a-positive lymphocytes were only in small number in each tissue core. The number of macrophages in the lungs correlated with the age of the pigs (*r* = 0.39, *P* < 0.05). Although statistical significance was not reached, cases with lower PPV2 Ct values tended to have higher numbers of macrophages (*r* = −0.23, *P* = 0.11) and T cells (*r* = −0.20, *P* = 0.17). With respect to the possible impact of PPV2 and other co-infected viruses on pneumonia, a stepwise multiple regression analysis was performed to determine which combination of PPV2, PRRSV, and IAV accounted for the greatest proportion of variance in macrophages. PPV2 was selected as a predictor variable with the highest contribution to the number of macrophages (*P* = 0.11), while IAV and PRRSV were eliminated.

### Metagenomic and Phylogenetic Analysis

Twenty qPCR-positive PPV2 samples that had no PCR history or were PCR negative for PRRSV, PCV2, and IAV were analyzed by metagenomic sequencing (Supplementary Table 2). One qPCR-positive PPV2 sample (no. 20804) that was PCR positive for PRRSV (Ct = 25.01) and IAV (Ct = 35.19) was analyzed by metagenomic sequencing as a positive internal control (Supplementary Table 1). Among them, PPV2 was detected in only two samples, both of which had qPCR Ct values of <18. Near-complete PPV2 genomes of 5,215 and 5,208 nucleotides in length were assembled, which were >99.7% identical to other PPV2 in GenBank. The remaining 18 samples had Ct values ranging from 21.3 to 33.6, suggesting that the sensitivity for PPV2 detection of our metagenomic sequencing protocol is Ct < 18. For seven of the 20 samples sequenced, no other recognized respiratory viral pathogens were detected.

**Figure 3:**
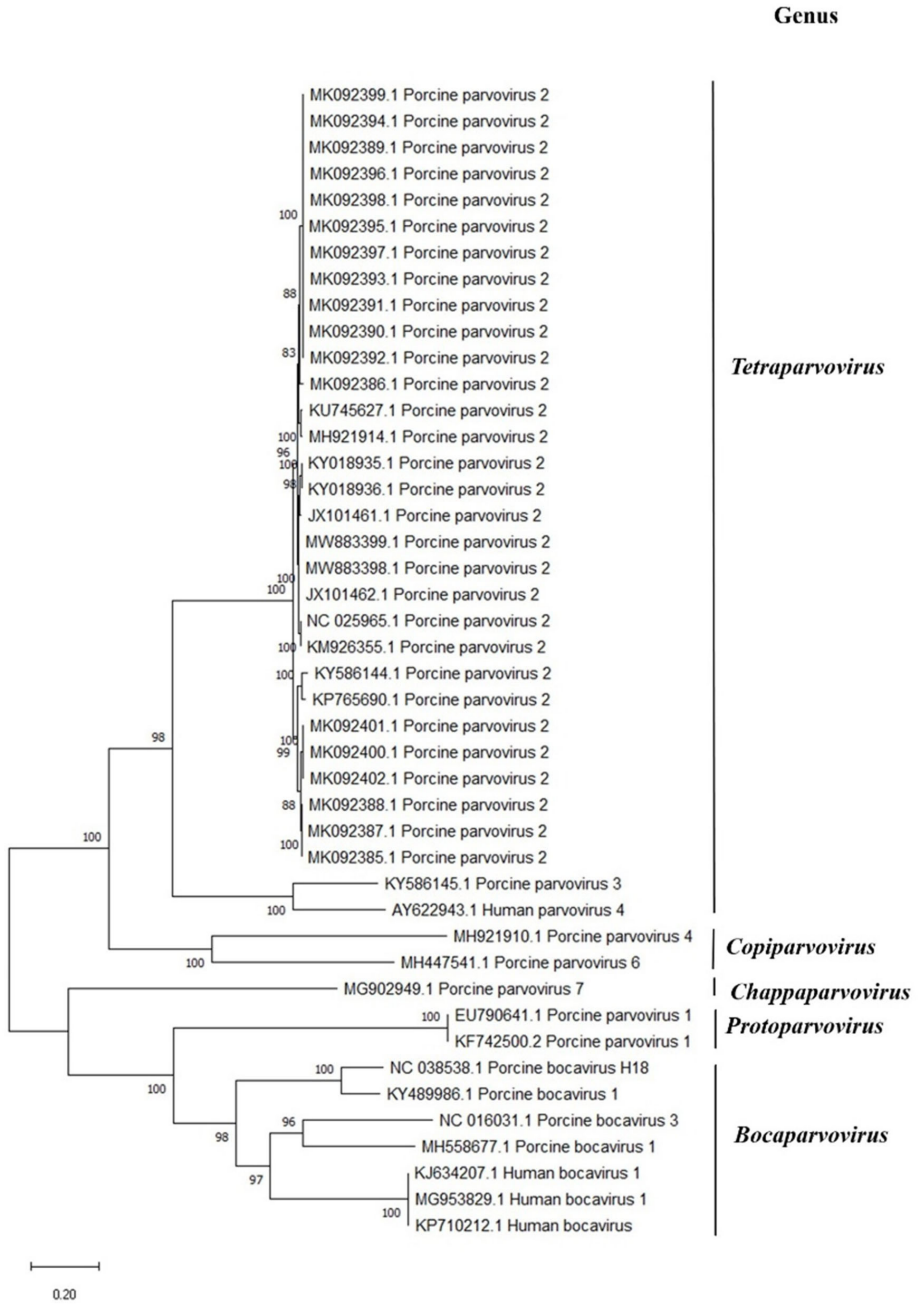
Phylogenetic analysis of members of the Parvovirinae subfamily in comparison to porcine parvovirus 2 (PPV2). The analysis involved 44 nucleotide sequences of vertebrate parvovirus with their GenBank accession numbers *marked in the tree*. The percentage of trees in which the associated taxa clustered together is shown *next to the branches*. Evolutionary history was inferred by using the maximum likelihood method and the Kimura two-parameter model. There were a total of 6,165 positions in the final dataset. Evolutionary analyses were conducted in MEGA X.

Consistent with previous studies, the PPV2 sequences from our study display a high level of similarity among the PPV2 strains worldwide (Figure 3) (9, 12).

### Characterization of PPV2 ISH Signals in Alveolar Macrophages

**Figure 4:**
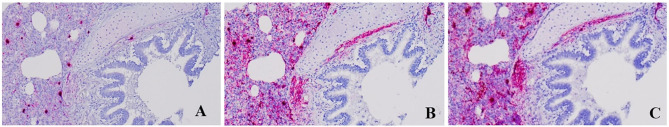
Detection of porcine parvovirus 2 (PPV2) by *in situ* hybridization (ISH ×200). The ISH signal of PPV2 was markedly reduced in formalin-fixed paraffin-embedded (FFPE) sections treated with DNase **(A)** compared to untreated FFPE sections **(C)**. There was no obvious difference between the FFPE sections treated with RNase **(B)** and the untreated FFPE sections **(C)**.

Compared to those in slides that had no treatment, the PPV2 ISH signals in DNase-treated slides were markedly reduced (Figure 4). However, strong intranuclear and intracytoplasmic signals of PPV2 were retained in the cells morphologically resembling alveolar macrophages and, occasionally, lymphocytes (Figure 4). Those treated with both DNase and RNase showed similar patterns to those treated with DNase alone. There was no obvious difference between the RNase-treated and non-treated slides in ISH, which, combined with the DNase treatment results, suggests that the majority of the ISH signals are due to probe hybridization with PPV2 ssDNA as opposed to PPV2 mRNA.

### Isolation of PPV2

Whether the inoculated PPV2 was removed or retained in the culture medium, the virus titer consistently declined, with increasing Ct values following each passage (Supplementary Table 3). Strong intracytoplasmic, but not intranuclear, signals of PPV2 were detected in the inoculated, unadsorbed alveolar macrophages by ISH for samples with a PPV2 qPCR Ct < 21. These samples also showed concurrent infections with IAV or PRRSV.

This suggests that PPV2 does not infect PAM cells *in vitro* under the conditions assayed and that the PPV2 detected by qPCR and ISH is due to phagocytosed genetic material.

## Discussion

Porcine parvovirus 2 is globally distributed, with frequent high prevalence in pigs (3). Despite occasional detection in clinical specimens, its etiologic significance is unknown (10, 12). Here, we identified PPV2 as highly prevalent in US pigs with respiratory disease, with PPV2 ISH staining mainly in alveolar macrophages in lungs with interstitial pneumonia. Interestingly, we found a higher prevalence of PPV2 (39%) than of the established porcine respiratory viral pathogens, PRRSV (32.9%), IAV (28.2%), and PCV2 (22.2%), in lung tissues from pigs with acute respiratory disease. There was a negative correlation between the Ct value of PPV2 and the age of pigs (*r* = −0.28, *P* < 0.05), with the highest prevalence detected in grow-finish pigs (63.6%) with respiratory disease. Similarly, other studies noted grow-finish pigs to have the highest prevalence of PPV2, suggesting that weaned, but not fully mature, pigs are targeted by PPV2 (7, 8, 12). Furthermore, the PPV2 genome sequences determined here demonstrated high similarity to the PPV2 strains from geographically and temporally diverse samples originating back to 2012.

Porcine respiratory disease complex is the result of host, environmental, and pathogen factors that often include multiple bacterial and viral co-infections, with PCV2, PRRSV, and IAV considered significant (14). While many studies have assessed the clinical outcomes of co-infection or superinfection, the interactions between microorganisms and their hosts and the underlying mechanisms remain unclear (14). It is not surprising that we found co-infections with PRRSV, IAV, PCV2, and *M. hyopneumoniae* in many of our cases. We hypothesized that multiple co-infections can worsen the clinical outcomes due to a potential synergistic effect on viral replication. In this study, however, PRRSV or IAV titers, as inferred by the PCR Ct values, were not proportionally higher in those cases with higher titers of PPV2. In addition, a review of the serial TMA sections showed that the location of the PPV2 signal rarely matched that of PRRSV or IAV, despite previous work showing that the monocyte/macrophage lineage is a common target of PRRSV and PPV2 infections (1, 13, 19). The lack of correlation in the Ct values of PPV2, IAV, and PRRSV, as well as the lack of co-localized viral signals by ISH and IHC, suggests that natural infections of PPV2 and other respiratory pathogens can be independent events in pig farms.

Porcine parvovirus 1 is a well-known cofactor triggering the development of PCVAD, including PWMS and PRDC (4, 5). Experimentally in PMWS, PPV1 DNA has been detected in tissue macrophages, including the lung (5, 20). Similarly, co-infection of PPV2 was frequently detected in pigs with PCVAD in the field (4). More recently, a retrospective study proposed a statistical model based on veterinary scores and latent class analysis and demonstrated a clustering cofactor association between PPV2 and PCV2, suggesting synergies in pathogenesis (7). In the present study, however, the prevalence and the titer of PCV2 were low based on qPCR. No obvious signal of the PCV2 antigen was detected in 99 TMA cores. Different from previous or retrospective studies, our observations reflect that, nowadays, PCVAD is well-controlled, at least in the United States where our samples originated, since the development and widespread usage of vaccine (21). On the other hand, high prevalence and titer of PPV2 were detected in our samples, with diffuse and intense PPV2 ISH signals in the lungs (Figures 1C–E). Interestingly, these images are similar to the IHC/ISH of PCV2 in PCVAD, in which abundant PCV2 genomes accumulated in alveolar macrophages, as well as in some lymphocytes and endothelial cells (5, 22). The germinal center of lymphoid follicles is demonstrated at early sites of PCV2 infection (15, 23). Similarly, we observed a strong positive signal of PPV2 in lymphoid tissues in PBLT (Figures 1D, 2D). Combined with their genetic similarity in terms of both being small, ssDNA viruses with tropisms for rapidly dividing cells, PCV2 and PPV2 may have similar pathogenesis. However, this hypothesis needs to be examined in further research.

Facing the highly complex pathogenesis of PRDC, we used high-throughput TMA with image analysis software (semi-quantitative IHC/ISH) and statistics to develop a mathematical model to investigate the significance of PPV2 infection. Both simple correlation and multiple regression analyses showed that higher levels of the PPV2 ISH signals tended to be associated with a higher number of macrophages in the lungs, suggesting a more severe pneumonia. This observation also aligned with that of previous and present studies that PPV2 had been mainly detected in macrophages (1). Traditionally, a *P* < 0.05 was set to reject the null hypothesis. However, as a guidance stated by the American Statistical Association in 2016, a *P* < 0.2 should be considered important in the interpretation of a large complex dataset, such as that of the present study (18). The statistical results here were addressed and further supported by pathology and metagenomic assays. The lack of statistical significance in our multiple regression model emphasized that, in addition to PPV2, some unidentified factors, such as age, bacterial infection, immune state, and individual variation, had dominant contributions to the development of PRDC.

As described in a previous PCV2 study, the high-throughput aspect of TMA has a clear benefit when compared to analyzing individual slides from each tissue (15). The small cylindrical tissue sections can be a concern for adequate representation, particularly when compared with qPCR on sensitivity. However, PPV2, as well as many other pathogens, is commonly identified in both clinically healthy and diseased pigs (2, 8–11). Demonstration of a high level of PPV2 in associated lesions is therefore important for the diagnosis of PPV2-associated pneumonia (4). In addition, our results showed that PCR is not an effective method to detecting PPV2 nucleic acid in FFPE samples (data not shown), probably due to the small tissue section or quality of the DNA extraction. In this study, all cases of PPV2 with Ct values <20 were detected in TMA cores. This observation led us to believe that TMA could be useful for large-scale retrospective and epidemiological studies.

Metagenomic sequencing provided an unbiased screening of pathogens in a small number of samples. We found no significant viral pathogens in over one-third of the samples analyzed. However, sensitivity is an intrinsic limitation of this methodology, as PPV2 was only detected in samples with low Ct values (<20). Additionally, our metagenomic sequencing workflow is intended to enrich for viral nucleic acids, thus lowering our confidence in bacterial identification. While not demonstrative of causation, the combination of qPCR and metagenomic sequencing identified seven cases where PPV2 alone, or with some bacterial co-infection (as per the result of routine bacterial culture; Supplementary Table 1), was present in tissues with marked pneumonia. Together, these results are suggestive of PPV2 having an etiologic role in PRDC.

Given that macrophages are the main cells responsible for the clearance of viruses in the bloodstream, the accumulation of PPV2 nucleic acid in the cytoplasm of alveolar macrophages may be from the phagocytosis of viremia or active PPV2 replication. While RNAscope^®^ probes are designed to specifically hybridize target mRNA, heat denaturation combined with the ssDNA genome of PPV2 may lead to probe hybridization to genomic DNA. To explore these possibilities, we treated slides with DNase to remove PPV2 DNA, intending to detect only the PPV2 mRNA signal. DNase treatment markedly reduced the PPV2 ISH signal, leaving strong intranuclear and intracytoplasmic signals in alveolar macrophages and in occasional lymphocytes, suggesting a possible PPV2 replication in PAMs. However, the dramatic decrease of the PPV2 signal following DNase treatment, combined with the minimal effect of RNase treatment, suggests that the vast majority of the PPV2 ISH signals are due to probe hybridization with genomic DNA. Other parvovirus studies have shown parvoviral DNA detection with ISH using the commercially available kit ACD RNAscope^®^, further emphasizing that the PPV2 signals detected in our study were mostly detecting PPV2 DNA (24, 25).

In this study, we investigated the natural infection of PPV2 in pigs with PRDC, which is affected by many known or unknown factors in the field. Ideally, experimental inoculation of PPV2 in pigs maintained in well-controlled laboratory conditions can be a model for clarifying the disease pathogenesis. However, given that many pigs infected with PPV2 alone were asymptomatic in the field, it is likely that pigs inoculated with PPV2 alone will develop minimal to mild clinical signs and pathological lesions, similar to the results of previous experimental infection of PPV1 in weaned pigs (8, 20). Therefore, obtaining high titer of infectious PPV2 stock and understanding the mechanism of PPV2 replication are critical for the reproduction of diseases in animals. Herein, *in vitro* cell culture and virus isolation were attempted. The intense ISH signals for PPV2 in alveolar macrophages suggested that PAM cells may support PPV2 replication. Other swine viruses, notably PPV1 and PRRSV, replicate in PAM cells, which are commonly used for PRRSV isolation (19, 26, 27). Our attempts to isolate PPV2 were unsuccessful; however, the samples with PRRSV or IAV concurrent infections maintained positive qPCR values for PPV2. Further investigations regarding the pathogenesis of PPV2 and other co-infections in cell culture are ongoing.

In conclusion, our study confirmed the high prevalence of PPV2 in diseased pigs and provided insights into its significance in PRDC. Detection of intensive and diffuse PPV2 ISH signals in interstitial pneumonia (Figures 1C–E), correlation between the PPV2 load and the number of macrophages, and the exclusion of other viral pathogens by metagenomic assay were evidences supporting PPV2 as one of the primary viral pathogens in the natural development of PRDC, particularly in weaned to finishing pigs. Further studies on the role of bacteria with PPV2 and PRDC will be important. Our initial work on viral isolation, as well as the similarities of PPV2 and PCV2, calls for continued work to isolate PPV2 and further investigate PPV2 in clinical disease with animal studies.

## Data Availability Statement

The datasets presented in this study can be found in online repositories. The names of the repository/repositories and accession number(s) can be found in the article/Supplementary Material.

## Ethics Statement

The animal study was reviewed and approved by South Dakota State University Institutional animal care and use committee 17-028A.

## Author Contributions

AN helped with the investigation and wrote the original draft. C-ML conceptualized and supervised the study, helped with the methodology, formal analysis, funding acquisition, wrote the original draft, and contributed to manuscript review and editing. BH conceptualized and supervised the study, helped with the methodology and funding acquisition, and contributed to manuscript review and editing. All authors contributed to the article and approved the submitted version.

## Conflict of Interest

The authors declare that the research was conducted in the absence of any commercial or financial relationships that could be construed as a potential conflict of interest.

## Publisher's Note

All claims expressed in this article are solely those of the authors and do not necessarily represent those of their affiliated organizations, or those of the publisher, the editors and the reviewers. Any product that may be evaluated in this article, or claim that may be made by its manufacturer, is not guaranteed or endorsed by the publisher.
