# Supplementary material for: Porcine Parvovirus 2 Is Predominantly Associated With Macrophages in Porcine Respiratory Disease Complex
Source: Front Vet Sci. 2021 Aug 13;8:726884. doi: 10.3389/fvets.2021.726884 (PMC8414833; doi:10.3389/fvets.2021.726884)
Supplement: Supplementary file 2 [file Table_2.DOCX]

|  | **Case number** | | | | | | | | | | | | | | | | | | | | |
| --- | --- | --- | --- | --- | --- | --- | --- | --- | --- | --- | --- | --- | --- | --- | --- | --- | --- | --- | --- | --- | --- |
| **Sample** | **18014** | **19246** | **18435** | **25485** | **25391** | **22374** | **23243** | **21515** | **22935** | **22936** | **23626** | **20343** | **951** | **20804^a^** | **20738** | **21755** | **21763** | **23207** | **10160** | **20388** | **22593** |
| **PPV2** | - | - | - | - | - | - | - | - | X | - | - | - | - | X | - | - | - | - | - | - | - |
| **PPV7** | X | - | - | - | - | - | - | - | - | - | - | X | - | X | - | - | - | X | X | - | - |
| **Rotavirus A** | - | - | - | X | - | - | - | - | X | - | - | - | - | - | - | - | - | - | - | - | - |
| **Rotavirus B** | - | - | - | X | X | - | - | - | - | - | X | - | - | - | - | - | - | - | - | - | - |
| **Rotavirus C** | - | - | - | X | - | - | - | - | - | - | - | - | - | - | - | - | - | - | - | - | - |
| **PCV2** | - | - | - | - | - | - | - | - | - | X^b^ | - | - | X^b^ | X^b^ | - | - | - | - | - | - | - |
| **Bastrovirus** | - | - | - | - | - | - | - | X | - | - | - | - | - | - | - | - | - | - | - | - | - |
| **PRRSV** | - | - | - | - | - | - | - | - | - | - | - | - | - | X | - | - | - | - | - | - | - |
| **Bocavirus** | - | - | - | - | - | - | - | - | - | - | - | - | - | - | X | - | - | - | - | - | - |
| **PHEV** | - | - | - | - | - | X | - | - | - | - | - | - | - | - | - | - | - | - | - | - | - |

**Supplementary Table 2.** Metagenomic sequencing of twenty-one samples quantitative PCR positive for porcine parvovirus 2 (PPV2) where (X) indicates sequence detected and (–) indicates sequence not detected.

PPV2: porcine parvovirus 2, PPV7: porcine parvovirus 7, PCV2: porcine circovirus type 2, PRRSV: porcine reproductive and respiratory syndrome virus, PHEV: porcine hemagglutinating encephalomyelitis virus

^a^ used as positive internal control.

^b^ PCV2 qPCR was not performed.
